# A longitudinal core curriculum to strengthen interprofessional competencies in German undergraduate nursing education: a mixed methods study

**DOI:** 10.1186/s12909-026-10273-z

**Published:** 2026-09-03

**Authors:** Tanja Lehnen, Frederike Lueth, Lisa Wolter, Jutta Busch, Laura Püschel, Miriam Leimer, Anne C. Rahn, Katrin Balzer, Wolfgang von Gahlen-Hoops

**Affiliations:** 1 Christian-Albrechts-University of Kiel, Faculty of Medicine at the University Hospital of Schleswig-Holstein (UKSH), Institute for General Medicine, Working Group Didactics of Nursing and Health Professions, Kiel, Schleswig-Holstein Germany; 2 University of Luebeck, Institute of Social Medicine and Epidemiology, Nursing Research Unit, Luebeck, Schleswig-Holstein Germany

**Keywords:** Interprofessional education, Healthcare, Core curriculum, Nursing education, Mixed methods, Interprofessional collaboration

## Abstract

**Aim:**

Interprofessional education is considered an important strategy for improving interprofessional collaboration in healthcare practice. The aim of the interEdu project was to develop a longitudinal core curriculum to strengthen interprofessional competencies in undergraduate nursing education.

**Methods:**

The study was conducted using a mixed methods design with qualitative and standardized interviews and evidence syntheses. The results were triangulated via joint displays and were discussed in an expert workshop. The curriculum was designed by the interprofessional project team based on these results.

**Results:**

The interEdu curriculum comprises nine chapters. The central element is a framework of interprofessional competencies, which forms the basis of competence descriptions that are formulated over three years of nursing education. It has a spiral structure and offers modules and learning units based on the situational principle.

In order to facilitate a transfer between the different learning venues, appropriate learning methods are recommended. These are learner-centered to support collaborative learning and self-directed learning.

**Conclusions:**

The interEdu curriculum will be used to longitudinally integrate interprofessional education into educational programs for nursing and other healthcare professions. In particular, the spiral structure and the interprofessional competencies formulated longitudinally over the whole undergraduate education cycle should contribute to the continuous development of competencies.

**Supplementary Information:**

The online version contains supplementary material available at https://doi.org/10.1186/s12909-026-10273-z.

## Background

Interprofessional collaboration in healthcare practice is considered a key component for addressing the primary challenges of an increased spectrum of diseases and demographic changes. Furthermore, interprofessional collaboration is considered to improve patient safety and quality of healthcare [1–3].

According to the World Health Organization (WHO) [4], one strategy for successful interprofessional collaboration in healthcare practice is the implementation of interprofessional education (IPE). IPE is defined as a learning environment where “two or more professions … learn with, from and about each other” [5] to develop competencies for interprofessional collaboration (referred to here as interprofessional competencies, or IPC) [6]. Competencies are understood as inner prerequisites (dispositions) that are individually acquired and developed, and which become visible when performed [7].

IPE can have a positive impact on learners’ attitudes, knowledge and behaviors relating to interprofessional collaboration (e.g., different roles and tasks of the professions involved) [8–12]. In a scoping review Patel et al. show that “IPE interventions can significantly enhance communication skills, thereby potentially reducing medical errors and improving patient outcomes” [13].

The sustainable implementation of interprofessional learning activities is a long-term process. IPE should begin during the undergraduate education of health professionals [14], continue longitudinally over the entire training and study period, and integrate the three learning venues (theory, practice, third learning venue). The development of IPC is a multi-stage process requiring time for learning and reflection processes. The initial focus should be on activities aimed at becoming acquainted with other professions and building confidence. Learners should be successively encouraged to combine the contributions of different professions in increasingly complex tasks in order to achieve the best possible quality of care for those who require it [8, 15]. To achieve a staggered and repetitive arrangement of IPE, a spiral-structured curriculum is required that enables the development and building of IPC as a process. A spiral curriculum is a didactic concept that aims to repeatedly address learning content in increasing complexity and depth throughout the entire educational program. In this way, learners repeatedly deal with and deepen the topics and content of the curriculum in order to enable a continuous development of competencies [16]. The concept of the spiral curriculum, as originally proposed by Bruner in 1960 [16] and widely used in health professions education [17], typically describes the gradual revisiting and deepening of content within a single learning environment, most often in the classroom. A cross-setting application of the principles of the spiral curriculum has not yet been systematically described in the existing literature on IPE curricula. Lindh Falk and colleagues (2024) stated that IPE often lasts only a few weeks, even within the context of clinical placements [18]. Overall, there are many initiatives, with a wide range of types and varying durations. However, there is a lack of spiral curricula that systematically integrate all three learning environments.

Existing international IPE curricula can be found, for example, in Sweden and Canada [19, 20]. A systematic review of frameworks and curricula conducted as part of the interEdu project [21, 22], however, found no-cross framework consensus on which compentencies constitute a sufficient interprofessional competency profile. None of the reviewed frameworks was developed for, or empirically validated in, the structurally distinct German undergraduate nursing education system.

In Germany, the Nursing Professions Act (*Pflegeberufegesetz*, PflBG) of 2020 defines, for the first time, competencies for IPC as a binding objective of undergraduate nursing education [23]. There are two pathways in German undergraduate nursing education. Whereas the most common pathway in Germany is three-year vocational training, academic training (three to four years) has also been firmly anchored in law since 2020. Both training pathways lead to recognition as a registered nurse; however, the typical roles and scopes of practice of vocationally versus academically trained nurses have so far been scarcely defined by law or other regulations [24].

According to legal requirements since 2020, 300 h must be set aside for vocational training to promote competencies for intraprofessional and interprofessional collaboration. In academic training, this is at the discretion of the universities. It is important to harmonize the objectives, content, and methods of the training programs to ensure comparable standards and enable a meaningful evaluation of learning outcomes. Currently there is no national curriculum for IPE existing as a reference framework for both pathways of undergraduate nursing education, the academic and the vocational erducation. To close this gap, the interEdu project (2022–2024) aimed to develop and pilot a longitudinal core curriculum for the training of IPC in undergraduate nursing education in Germany, including a design phase (20 months) and a pilot phase (16 months) [25].

As a main result, this paper describes the design phase and presents key elements of the longitudinal core curriculum for undergraduate nursing education, as per the recommendations for reporting (see CONFERD-HP )Recommendations for Reporting COmpeteNcy FramEwoRk Development in Health Professions) Checklist [26] (Additional file 1).

## Methods

### Study design

**Fig. 1:**
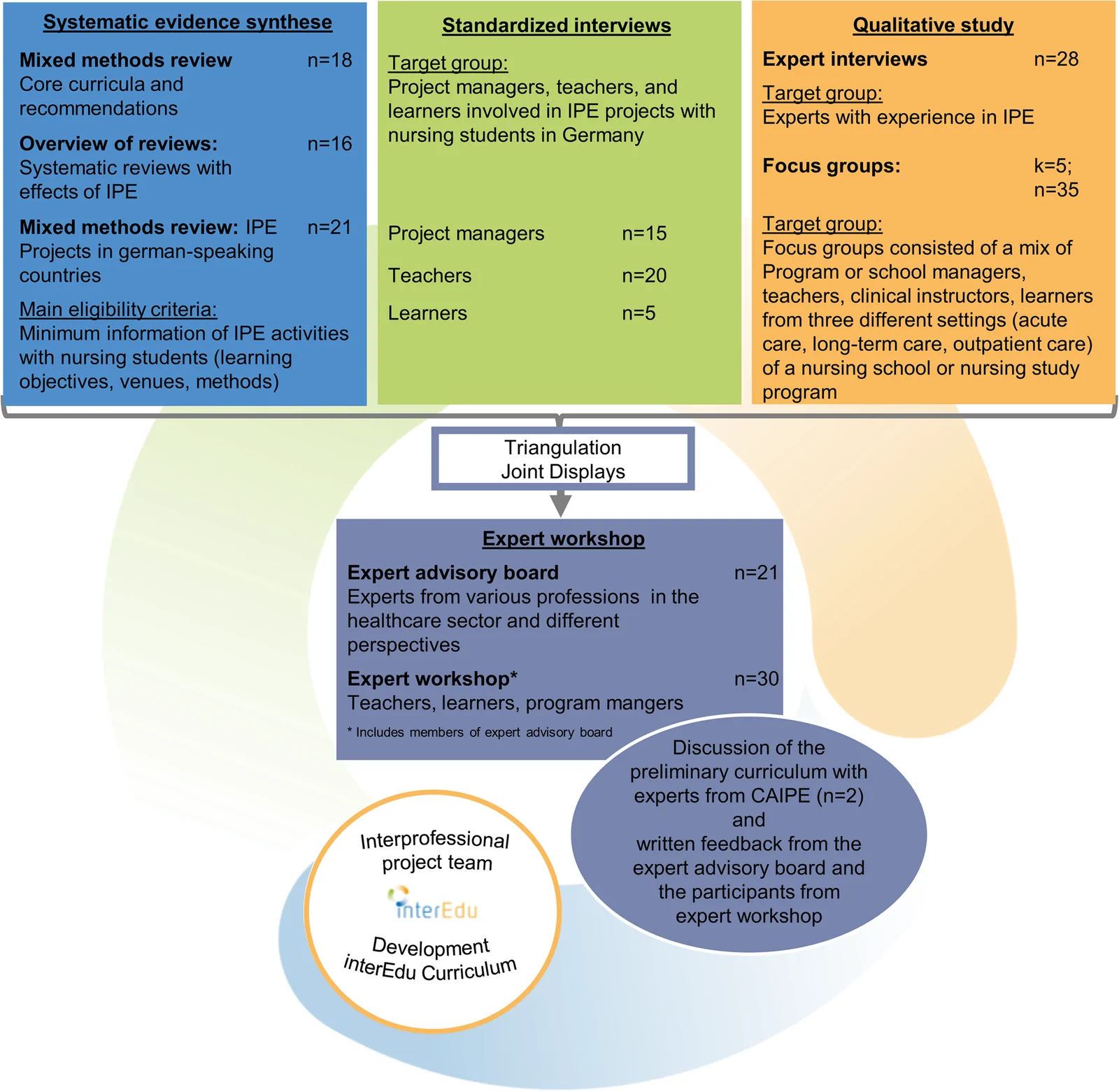
Curriculum design process

A convergent mixed methods approach [27] (see Fig. 1, Additional file 2, Additional file 3) guided the design phase (curriculum development), combining the following quantitative and qualitative data sources: evidence syntheses framed by rapid review methodology [28], standardized interviews, and qualitative interviews (focus groups and expert interviews). These data sources were triangulated using joint displays to identify core elements of the curriculum. These were discussed with experts in a structured workshop. Based on the workshop findings, the interprofessional project team conceptualized the preliminary core curriculum. The project team included professionals, teachers, and researchers with backgrounds in nursing, speech therapy, and physiotherapy. Throughout the design phase, the project team iteratively reflected on the intermediate results from the single strands. The design phase of this study was conducted with the approval of the Ethics Committee of the University of Luebeck (File number: 22–156).

### Data sources and triangulation

Figure 1 shows key characteristics of the three main data sources combined in the design process: systematic evidence syntheses consisting of one mixed methods review of existing interprofessional core curricula and frameworks, one overview of reviews on the effects of IPE activities and one mixed methods review specifically summarizing results of IPE projects in Germany, a qualitative study comprising expert interviews and focus groups with program or school managers, teachers, clinical instructors and students to explore their IPE experiences and expectations to an IPE core curriculum, and standardized interviews with project managers, teachers, and (previous) learners involved in completed/existing IPE projects in German nursing education. The data from these sources were analyzed separately, and the results are summarized in Additional file 3. The results were triangulated using joint displays [29] to identify preliminary core elements for the curriculum. To analyze the various data sources, side-by-side joint displays, consolidated presentations in the form of a table were chosen [30]. To synthesize the findings from the evidence synthesis, the standardized interviews, and the qualitative interviews, six thematic side-by-side joint displays were conducted based on the design principles for curricula of nursing education (e.g [31, 32]), with the following headings: learning objectives and interprofessional competencies, learning venues, learning methods and social forms, participants (learners, instructors, tutors), evaluation methods, contextual factors for the implementation. Findings from all methods (evidence syntheses, standardized interviews, qualiative interviews) were mapped by learning venue (practice, nursing school/university, and third learning venue/Skills Lab), type of education (academic, vocational, comprehensive, unclassifiable), and year of education (1st, 2nd, and 3rd). Important dimensions (e.g. group work as teaching-learning format) were identified and informed by qualitative interview quotations. The integrated results formed the basis for curriculum development for each year of training and vocational and academic nursing education.

### Expert advisory board and workshop

The triangulated results were the basis for the expert workshop (*n* = 30) in February 2023. It was conducted to discuss, adapt, and specify the preliminary core elements of the longitudinal core curriculum for the training of IPC in undergraduate nursing education.

Participants were persons of the cooperating institutions (nursing schools and universities implementing the curriculum in the second project phase) and members of the expert advisory board (*n* = 21), who were recruited at the beginning of the project and comprised national and international members (Switzerland) with expertise in IPE and collaboration from different learning venues (theory, practice, skills lab/simulation) having different roles and professions (Table 1). The members were recruited via e-mail with detailed information regarding the project aims and methods.

**Table 1:** Characteristics of the experts involved in the advisory board and the workshop

	Expert advisory board (*n*=21)	Expert workshop (*n*=30a)	Total (*n*=32)
Setting			
Vocational training	3	10	10
Higher education	8	10	10
Healthcare practice	4	4	4
Other	6^b^	6^c^	8
Role (multiple roles per member possible)			
Student/learner	0	3	3
Teacher	14	16	18
School or program manager	9	8	10
Researcher	11	12	14
Instructor/perceptor/education coordinator in healthcare practice	4	4	4
Other coordinating roles	3	6	6
Patient and/or caregiver representative	2	2	2
Profession			
Nursing	8	13	13
Medicine	2	3	3
Physiotherapy	3	4	4
Occupational therapy	1	1	1
Speech therapy	1	1	1
Interprofessional role	4	2	2
Not applicable	2	3	5
Country			
Germany	17	28	28
Other german-speaking country	2	2	2
United Kingdom	2	0	2

^a^Including 19 members of the advisory board

^b^Including *n*=2 patient representatives, *n*=2 members of the management team of the Centre for the Advancement of Interprofessional Education (CAIPE), *n*=2 working in network organizations promoting nursing education

^c^Including *n*=2 patient representatives, *n*=2 working in network organizations promoting nursing education, =2 working in the Federal Institute for Vocational Education and Training (Bundesinstitut für Berufsbildung, BIBB), Division of nursing professions

The workshop aimed to interpret the empirical findings, synthesize them through discussion, and reach a consensus on the key elements of the curriculum. This represents a qualitative approach, consensus development approach rather than a Delphi process, as consensus was achieved through face-to-face discussion instead of anonymous iterative surveys [33].

The workshop consisted of two phases: First, the results of the data analysis and the triangulation were presented; second, five small groups were formed for in-depth discussion of these results and identification of central implications for the core curriculum, e.g. regarding learning objectives, topics, didactic approaches, and learning venues. Three groups focused on vocational training and two groups on academic training, with each group addressing the first, second or third year of education, respectively (for higher education, the first two years had to be combined in the workshop due to pragmatic reasons). The groups were composed heterogeneously so that teachers, learners, and managers were always represented. Each small working group discussion was moderated and facilitated by one member of the project team using creative techniques (Miro^©^ https://miro.com/de/). Results were documented and narratively summarized, stratified vocational or academic nursing education and year of training (Additional file 3).

### Development

Based on the outcome of the expert workshop, the preliminary curriculum was developed in multiple steps: First, the project team discussed the workshop results, taking into account relevant findings from the foregoing data triangulation, and outlined the structure of the curriculum. Then, assigned members of the project team drafted single chapters, again integrating the workshop results with the empirical evidence of the triangulated data sources. At this stage, also prototype learning units (LU) for vocational training and teaching modules for higher education were designed based on the learning objectives, topics and further didactic implications suggested by the workshop participants. Throughout this development phase, interim results were iteratively discussed within the whole project team.

Once the curriculum was completed, it was forwarded to the members of the expert advisory board for critical comments and suggestions. In addition, a discussion of the preliminary curriculum took place with two members of CAIPE (Centre for the Advancement of Interprofessional Education). After this feedback period, the curriculum was revised on the basis of the comments and suggestions received.

## Results

### Overview of the curriculum

The interEdu curriculum addresses persons primarily involved in the development and implementation of curricula. Essentially, these are teachers, learners, instructors, program managers, and persons responsible for the coordination of theoretical and/or practical training.

It is also aimed at people in practical training institutions, political decision-makers, and professional associations.

**Fig. 2:**
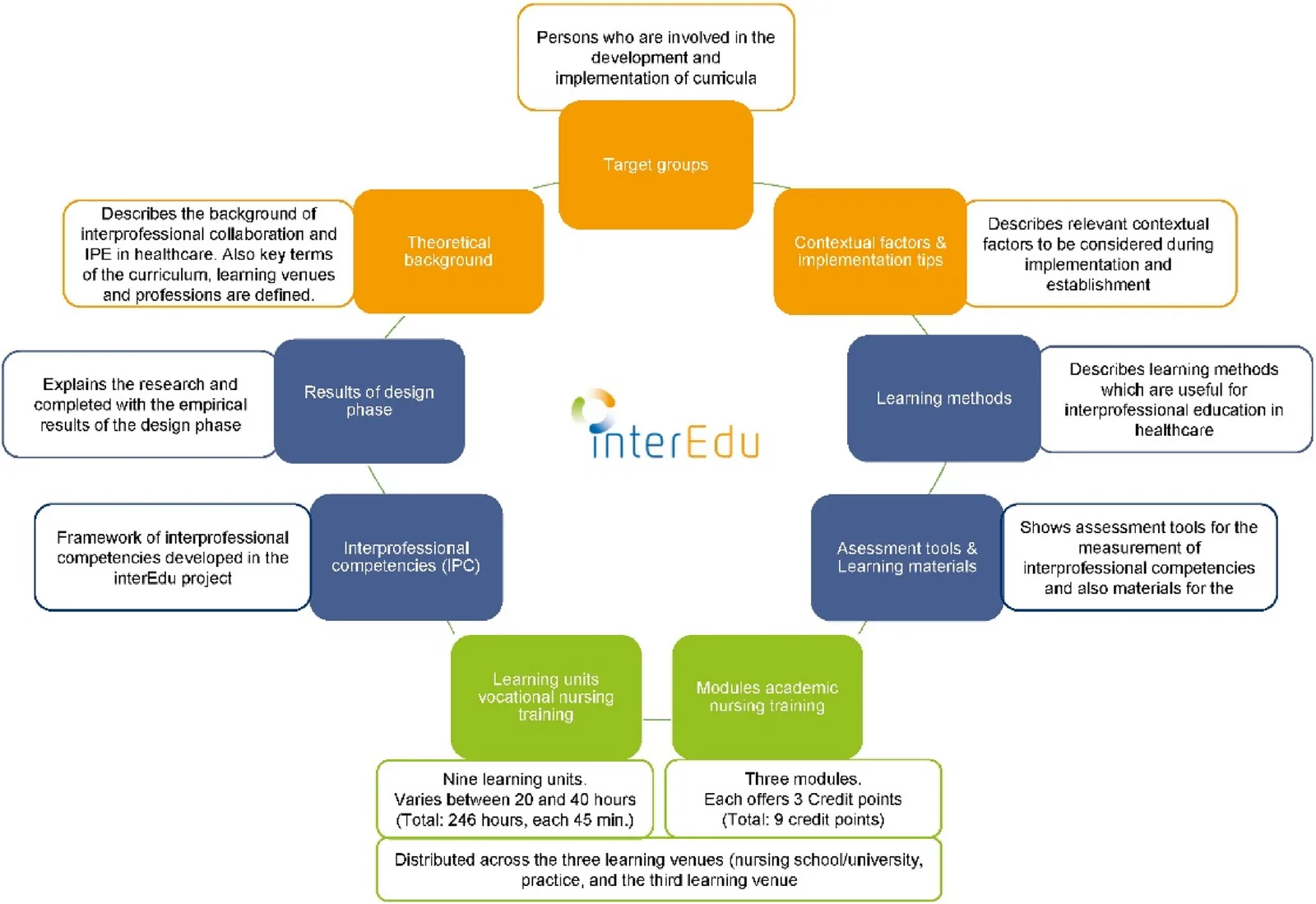
Main chapters

**Fig. 3:**
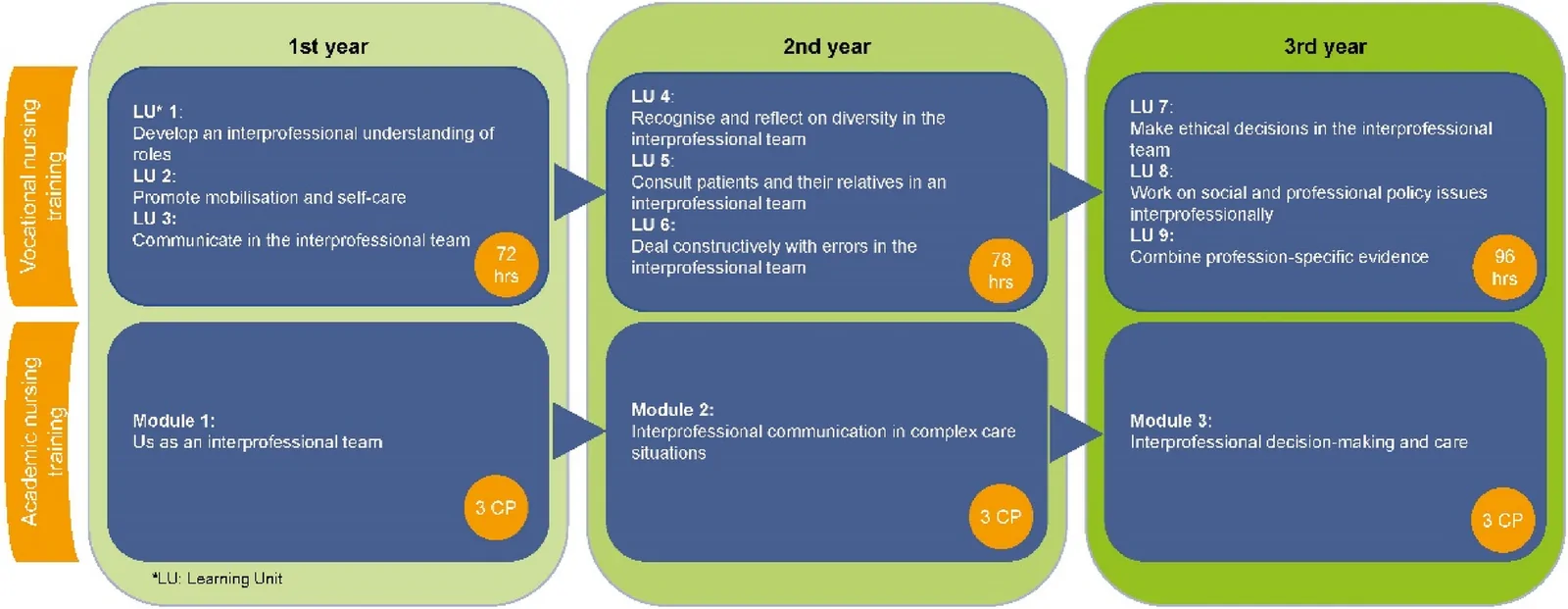
Learning units and modules

A joint curriculum was designed for vocational and academic nursing training, which consists of nine chapters. The content of these chapters is briefly summarized in Fig. 2. In order to meet the requirements of the respective academic and vocational training pathways, differentiations were made in the IPC and learning units (LU)/modules, which are a central part of the interEdu Curriculum. Nine LU were developed for vocational training, three for each year; one module per year was developed for academic training (Fig. 3).

The LU/modules of the interEdu curriculum provide details regarding scope, content, and learning methods in IPE, learning materials (e.g., simulation scenarios), assessment tools, and contextual factors (e.g., different frameworks and curricula) that should be taken into account for implementation.

### Insight into the curriculum – competence model

The essential requirements for a core curriculum to support the development of IPC is a spiral curriculum that offers learning opportunities over the entire duration of the educational program and promotes the continuous development of IPC. Because the spiral structure of the curriculum enables continuous competency development throughout the educational pathway, for each target domain of competencies (as described below) objectives were formulated that increase with each year of education.

**Fig. 4:**
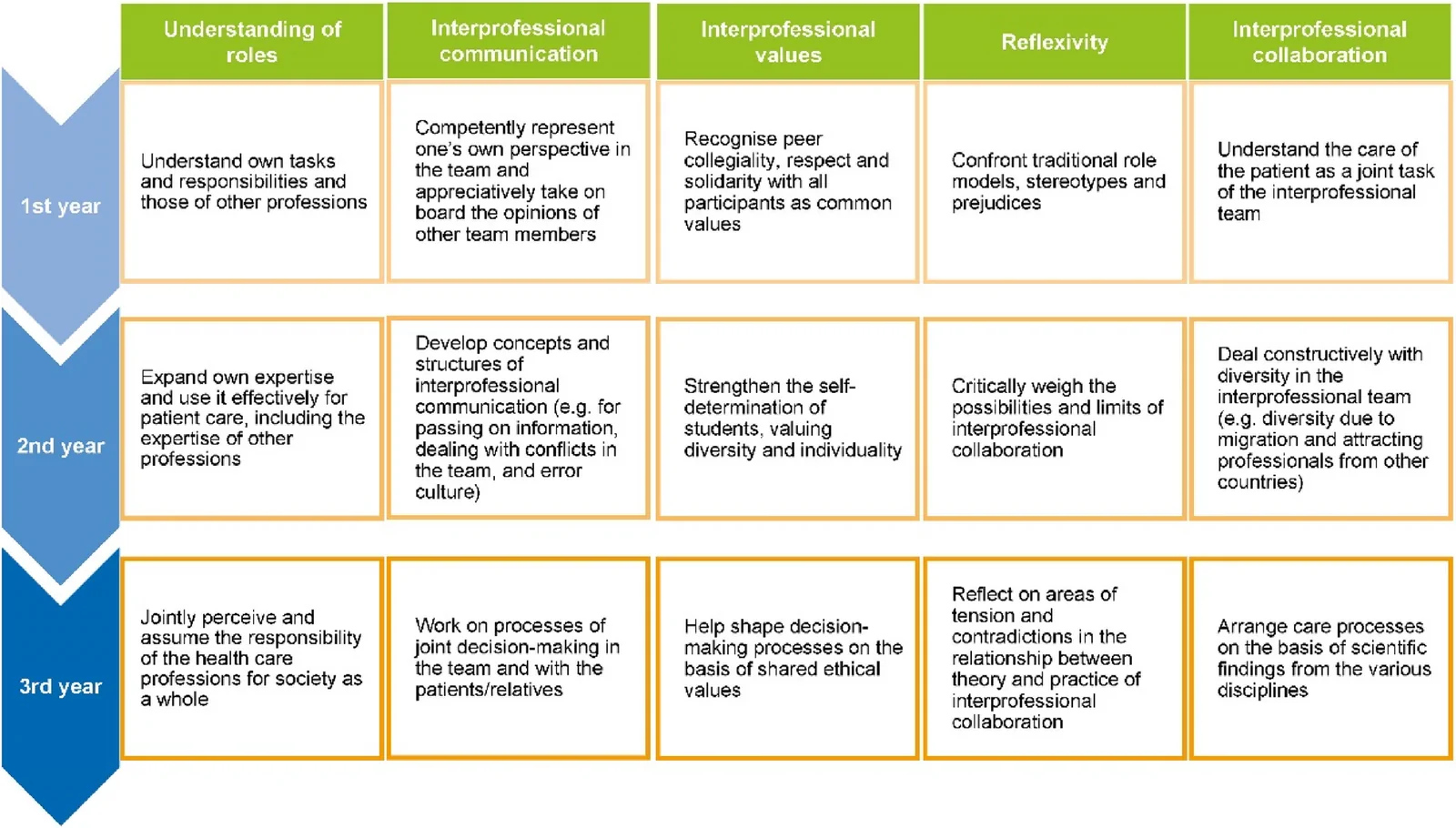
General interprofessional educational objectives of the interEdu curriculum

The objectives are differentiated into interprofessional educational objectives (Fig. 4) and more detailed IPC. Educational objectives describe reflective competencies that go beyond professional competence, in order to develop a critical personality and identity that is achieved through an examination of social reality, which is often characterized by contradictions. These contradictions can arise, for example, when decisions must be made in a situation of conflict between autonomy and care [31].

Building on the educational objectives, the IPC were formulated in accordance with the domains for interprofessional collaboration. Given the large number of existing competence frameworks identified in the design phase (evidence synthesis [22]), the specific IPC model for the interEdu curriculum draws on three complementary frameworks capturing key domains that are not fully represented by any single framework [4, 34, 35].

The first framework that draws the new developed model is the “Framework for Interprofessional Education and Collaborative Practice,” which the World Health Organization developed in 2010 as a health policy guideline for interprofessional education and practice in the health professions, with a focus on collaboration with patients and their families [4]. Second, the “Guidelines for Interprofessional Education” [34] from the Center for the Advancement of Interprofessional Education (CAIPE) were used, which emphasize that the goal of interprofessional education is to ensure comprehensive, high-quality care through collaboration—which goes beyond mere cooperation. The goal of high-quality care is also reflected in the third selected framework. In the “International Consensus Statement on the Assessment of Interprofessional Learning Outcomes” [35], mutual understanding of roles, shared values, and effective and respectful communication are just as fundamental as critical reflection. An important consideration to develop an new model was that greater emphasis should be placed on the person-centered domain. Person-centred care is an approach that considers patients’ values and preferences and is based on a partnership between patients and healthcare professionals. It is important because it is desired by patients and their families and is promoted by the World Health Organization. It has also been shown that person-centred care improves the quality of care [36].

**Fig. 5:**
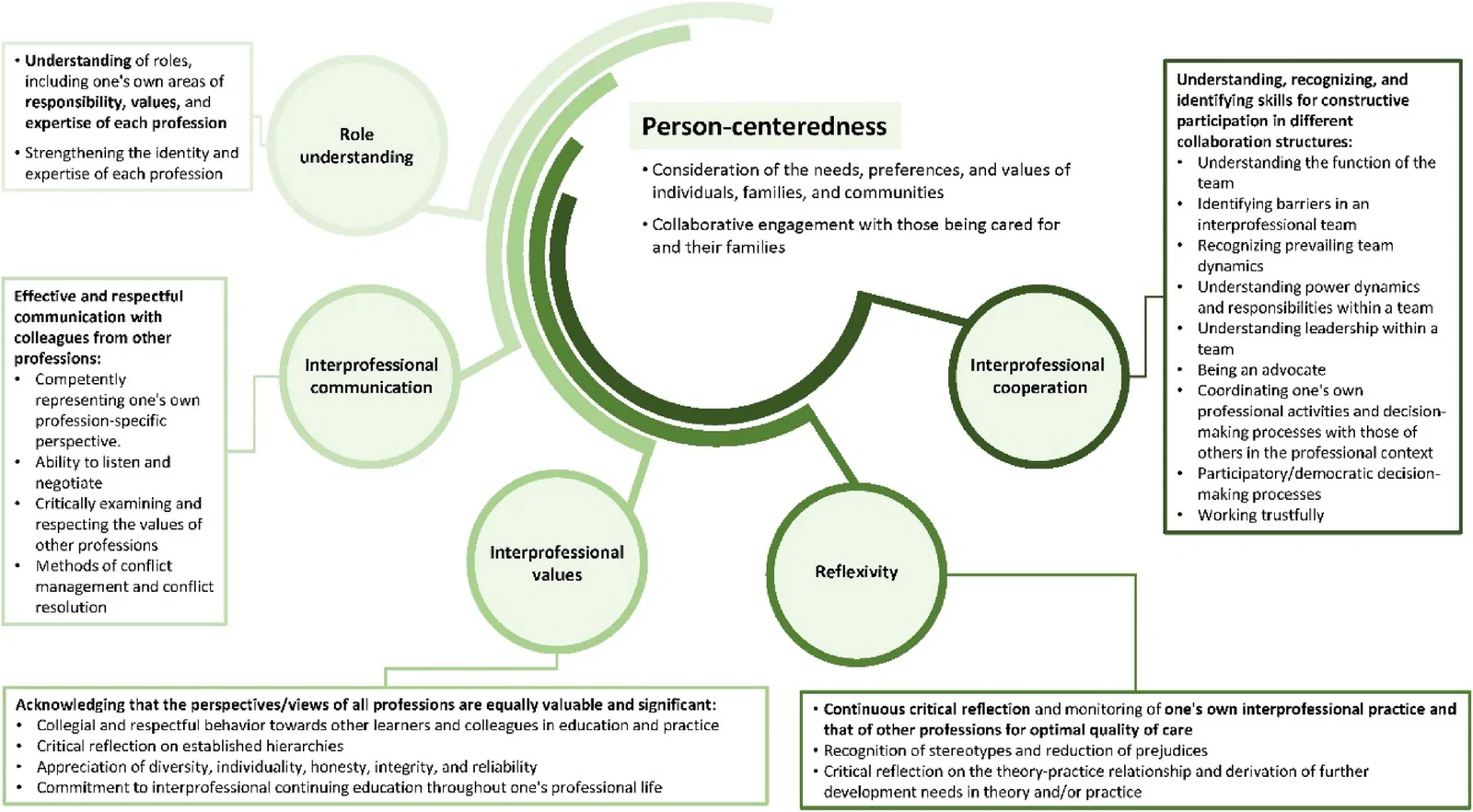
Domains of interprofessional competencies

Core of the new model are competencies for person-centred care, with five inter-related domains of IPC contributing to this nucleus: role understanding, interprofessional communication, interprofessional values, reflexivity, and interprofessional collaboration (Fig. 5). The educational objectives and IPC are formulated in stages for the three years of training, are interlinked with each other, and are characterized by increasing complexity and educational content over the three years of training.

Although both curricula, the modules for the academic and the LU for the vocational nursing education, are based on the same framework for core competencies, the academic nursing curriculum differs from the vocational curriculum in two key ways. First, it includes additional scientific competencies that enable students to identify, critically evaluate and apply research findings to shape clinical practice. Second, it prepares graduates to take responsibility for the assessment, coordination, and management of highly complex nursing situations characterized by multimorbidity, diagnostic uncertainty, rapidly changing patient conditions, and the need for advanced clinical reasoning. Thus, the educational objectives of the Interedu modules for academic education target distinct competences in person-centered decision-making processes with multiple involved parties, case management and care coordination and evaluation and furtherdevelopment of IPC in healthcare practice. The competencies in the LU for vocational training focus on competencies required to provide safe, person-centered care in routine and complex care situations.

### Insight into the curriculum – learning units and modules

In addition to the spiral structure described above, all three learning venues must be taken into account. This cross-venue application of spiral curriculum principles, as opposed to within-venue content spiraling alone has, to our knowledge, not been systematically described in the existing IPE curriculum literature and may represent a transferable contribution for other multi-site training contexts. The LU/modules contain classroom-based teaching and self-directed and integrated practical learning hours. For classroom-based teaching, theoretical and practical lessons are designed using pedagogical-didactic concepts and versatile learning strategies. Here, competencies are developed, consolidated, deepened, and expanded in the practical learning venue. This is achieved through guided and supervised, yet increasingly independent, application and reflection according to the training year. The aim is to enable learners to learn and act effectively, i.e., to translate theoretical and communicative competencies into concrete professional action: for example, applying interprofessional communication skills learned through role-play (LU 3) to a real interprofessional handover situation in clinical practice [37, 38]. The LU/modules are based on the situation principle, which states that in the development of curricula for vocational education and training, theoretical content should be related to empirical situations or cases. In addition to the situational reference, a scientific orientation must be incorporated [39, 40]. The interEdu curriculum therefore recommends methods in the individual modules/LU to promote the transfer between the learning venues. One example is an observation assignment (LU 6), in which students describe an error situation they experienced in clinical practice. These experiences will be discussed in nursing school and ultimately presented during a poster session (Gallery Walk). The results from the poster session can be used for assessment. An example of both an LU and module is provided in Additional file 4. The methods were chosen based on the triangulated results from the design phase. These findings suggest that simulation-based education (SBE) and case studies are considered particularly promising for supporting transfer, in addition to other learning methods, such as learning transfer exercises, which are worked on in practice and whose results are taken up again in theory after this. Furthermore, the design phase revealed a preference for methods that promote mutual exchange. SBE therefore offers a suitable learning environment, as learning generally takes place in small groups Of course, it is essential to ensure the interprofessional composition of the learning groups, and this also applies to the other methods recommended in each module/LU.

## Discussion

The interEdu curriculum is the first framework in Germany for the development of IPC for both nursing training pathways – vocational and academic – in undergraduate nursing education.

Based on empirical and theoretical foundations, it was designed in an iterative and participatory process. It is also intended to serve as a prospective template for other professions in the healthcare sector in order to integrate IPE into undergraduate programs sustainably.

The curriculum developed has the character of a recommendation. The scope, content and learning methods can be flexibly adapted to the respective conditions, e.g. previous knowledge or professions of the learners. This flexibility is intended to facilitate integration into existing curricula and thus support the consolidation of IPE as called for by Kaap-Fröhlich et al. [41]. The educational objectives and competencies for this curriculum were formulated on the basis of empirical and theoretical findings, providing competence descriptions for interprofessional collaboration for academic and vocational nursing training that take both training pathways into account, yet make the differences clear. The extent to which IPC are achieved through the curriculum will be explored in the pilot phase.

A spiral structure was chosen in order to achieve repetition and a continuous deepening of the content. It is also intended to achieve early integration of IPE in the course of the training, which is recommended [11].

The concept of the spiral curriculum [16, 17] typically describes the gradual revisiting and deepening of content within a single learning environment. The present interEdu curriculum extends this principle beyond mere content progression: During the three phases of training, competencies are not only revisited with increasing complexity but are also systematically addressed in three different learning settings: at the nursing school/university, in clinical practice, and in a third learning setting that combines structured practical and theoretical elements (e.g., skills lab, simulation) [42]. Learners therefore neither engage with the same competency areas in a linear sequence limited to a single context nor deepen their understanding in this way; rather, they do so across multiple, nonlinear pathways in various learning contexts, which enables repeated application of the competencies under conditions of increasing realism and complexity. Thus, the present interEdu curriculum describes an expanded, cross-learning-site application of the principles of the spiral curriculum. This has not yet been systematically described in the existing literature on IPE curricula and could represent a transferable model for other multi-site training contexts.

The data collected as part of the interEdu project also underline the early integration into existing curricula. According to the WHO, a central goal of IPE is to improve the quality of care practice, and suitable methods are required for the transfer of IPC into professional practice [4, 41].

It is important that learning venues are interlinked in terms of content and methodology, and that they complement each other in a meaningful way to support the transfer of learning content [43]. In order to promote transfer of learning, all three learning venues are taken into account in the modules/LU of the interEdu curriculum. Recommendations for transfer tasks as well as reflection tasks are described, which should contribute to a successful transfer. Various methods for promoting transfer are recommended. In addition, interlinked theoretical and practical content is considered relevant for promoting transfer, and the joint coordination of all persons involved in the training was taken into account in the design of the interEdu curriculum during data collection, as this included teachers of theory and practice as well as learners.

In order to promote IPC, the interEdu curriculum often recommends learning methods that enable learning in small groups and in which a high degree of time for exchange and joint work is to be achieved. This is also recommended by Diggele et al. (2020), who stated that small groups enable close interaction and promote the active participation of all learners as well as flexibly structured learning. Another advantage of small groups is the promotion of communication skills [9]. As interprofessional communication is a domain of interprofessional skills, this is an important aspect that needs to be considered in the methodological design.

The development of the curriculum has limitations. The expert advisory board accompanied the development process of the interEdu curriculum in a participatory approach that also incorporated international perspectives. Nevertheless, individual groups were underrepresented; learners and instructors, for example, were only represented in small numbers.

Also, the curriculum, especially the prototype LU/modules, have a strong focus on acute care situations. This is particularly evident in the causes for action and the case studies. This is probably due to a certain dominance of the acute care setting in reviewed IPE reviews and projects and in in the selection of the expert advisory board. Attention was paid to different perspectives (e.g., teachers, learners, managers, professions), but not to experiences in specific care settings. In future revisions of the curriculum, the scope of the curriculum should be expanded to other care settings, especially primary and long-term care, and research on IPE in these areas should be intensified.

In the qualitative results of the conception phase, it was noted several times that an interprofessional team would be required for the development of a curriculum for interprofessional IPC training. Although the interEdu project team covered different health professions, it should be noted that not all of them (particularly medical doctors) were represented. However, this deficit was compensated for by the expert advisory board within the project.

## Conclusion

The interEdu curriculum should be used to integrate IPE longitudinally into the educational programs for undergraduate nursing education, and is also intended to serve as a template for the integration of IPE into other healthcare professions. The spiral structure and the IPC formulated longitudinally over three years contribute to the continuous development of competencies. A pilot phase in which the curriculum is implemented in universities and nursing schools will show the extent to which this can be achieved.

The curriculum is intended for undergraduate nursing education to firmly establish IPE across the board and in the long term, and can be additionally implemented in other education and training.

## Supplementary Information

Additional file 1.

Additional file 2.

Additional file 3.

Additional file 4.

## Data Availability

The data supporting the findings of this study are available within the paper and its Supplementary Information.

## Abbreviations

BIBB: Federal Institute for Vocational Education and Training (in german: Bundesinstitut für Berufsbildung)
CAIPE: Centre for the Advancement of Interprofessional Education
CONFERD-HP: Recommendations for Reporting COmpeteNcy FramEwoRk
interEdu: Project title
IPC: Interprofessional competencies
IPE: Interprofessional education
LU: Learning unit
PflBG: Nursing Profession Act (in german: Pflegeberufegesetz)
SBE: Simulation-based education
