# Supplementary material for: A longitudinal core curriculum to strengthen interprofessional competencies in German undergraduate nursing education: a mixed methods study
Source: BMC Med Educ. 2026 Sep 3;26:1449. doi: 10.1186/s12909-026-10273-z (PMC13560283; doi:10.1186/s12909-026-10273-z)
Supplement: Supplementary file 1 [file 12909_2026_10273_MOESM1_ESM.docx]

**CONFERD-HP Checklist**

| **Section/topic** | **Item** | **Checklist item** | **Location**  **where item is reported** |
| --- | --- | --- | --- |
| **Title and abstract** | | |  |
| Title | **1a** | Identification as a competency framework in the title. | Title |
|  | **1b** | Identification of intended profession and level of practice/stage of training in title. | Title |
| Structured abstract/summary | **2** | Structured summary that includes intended user(s) and use(s) of the framework, the purpose of the framework, the development process and methods used. | Abstract  (User(s)) Results |
| Definition(s) | **3** | Defined or referenced definitions for competence, competency and other key terms used to promote understanding of the framework. | Background |
| **Framework development** | | |  |
| Rationale and justification | **4** | Description of rationale and justification for the development of the framework including supportive references where possible. | Background |
| Purpose and use | **5a** | Description of the purpose of the framework. | Background Results |
|  | **5b** | Description of the intended use(s) of the framework. | Results |
|  | **5c** | Description of the intended user(s) of the framework. | Results |
| Developer group | **6** | Description of the qualifications and expertise of those leading the development of the framework. | Methods |
| Oversight/governance group | **7** | Description of the group that had oversight of the framework, the purpose and expertise of the group members, how they reviewed the work and/or contributed to the development. | Methods |
| Theoretical/conceptual  approach(es) | **8** | Description of theoretical/conceptual approach(es) used to develop the framework including  references and rationale for their use. | Results |
| **Development process** | | |  |
| Process and methods | **9a** | Description of each step of the development process. | Methods |
|  | **9b** | Description of how existing literature was gathered and used to inform the competency framework development. Provide a list of references used. |  |
|  | **9c** | Description of all methods used throughout the development process including associated reference(s) and details of any modifications. | Methods |
| End-user contributions | **10** | Description of all stakeholders, including end users of both the framework (e.g., the professional group consulted) and the services (e.g., patients/consumers and other healthcare professionals) who contributed to the development process, how they were selected (with considerations of equity,  diversity and inclusion), and how they participated. | Methods, Results |
| Ethics | **11** | Description of ethical considerations and approvals obtained where applicable. | Methods |
| **Evaluation and implementation** | | |  |
| Evaluation | **12** | Description of the approach for evaluating the draft competency framework, including how feedback from stakeholders was gathered and used. | Methods |
| Implementation | **13** | Suggestion for how the framework should be implemented and in what settings. | Background  Conclusion |
| **Funding and COI** | | |  |
| Funding | **14** | Description of all funding sources and other support received for the development of the framework and the role of the funder(s). | Funding |
| Conflict of interest | **15** | Description of how conflicts of interest were considered and managed in the development process. | Declaration |

Adapted from: Batt A, Tavares W, Horsley T, Rich J, Williams B, and the CONFERD-HP Collaborators. ‘CONFERD-HP: Recommendations for Reporting COmpeteNcy FramEwoRk Development in Health Professions’. British Journal of Surgery, 22 November 2022, znac394. https://doi.org/10.1093/bjs/znac394. For more information visit conferd-guideline.org. v221125
