# Supplementary material for: A longitudinal core curriculum to strengthen interprofessional competencies in German undergraduate nursing education: a mixed methods study
Source: BMC Med Educ. 2026 Sep 3;26:1449. doi: 10.1186/s12909-026-10273-z (PMC13560283; doi:10.1186/s12909-026-10273-z)
Supplement: Supplementary file 2 [file 12909_2026_10273_MOESM2_ESM.docx]

Additional file 2: Data collection methods of the design phase

|  | **Evidence syntheses [1]** | **Standardized interviews** | **Qualitative interviews** |
| --- | --- | --- | --- |
| **Aim** | - Question 1 (Q1): Summarize (inter)national recommendations and frameworks for IPE learning activities (scope of learning activities: min. 18 months) - Question 2 (Q2): Summarize the results of student-, teacher-, patient-relevant outcomes of IPE learning activities - Question 3 (Q3): Identify characteristics, effects, and contextual factors of IPE projects in German-speaking regions (scope of projects: min. 15 hours teaching) | - Identify characteristics, effects, and contextual factors of previous IPE projects (incl. nursing students) in Germany | - Identify relevant aspects (IPC, current practice in the design of IPE, and relevant contextual factors that can influence IPE) for the design of the curriculum to promote IPC. |
| **Target group/ eligibility criteria** | - Population: Undergraduate nursing students, teachers, tutors, supervisors and instructors - Intervention: IPE primarily framed or co-framed in the context of nursing education - Outcomes (Q2): Effects on Kirkpatrick levels 2–4 [2] (measured using quantitative methods) must be reported - Design: - Q1: Recommendations, frameworks, core curricula, guidelines - Q2: Systematic evidence syntheses - Q3: Primary studies of IPE projects in German-speaking regions | - Project managers, teachers, and learners involved in IPE projects with nursing students in Germany | - Expert interview: Experts with experience in IPE - Focus group: Program or school managers, teachers, clinical instructors, learners from three different settings (acute care, long-term care, outpatient care) of a nursing school or nursing study program |
| **Data collection methods** | - Literature search using five databases (e.g. MEDLINE), back-tracking and additional search: - Q1: Portals of organizations (e.g., NEXUS, WHO) - Q2: BEME - Q3: Websites (e.g., Robert Bosch Foundation, Federal Ministry of Education and Research), Google Scholar - Title and abstract screening: One person screened and a second person cross-checked (first 20% screened separately by two persons) - Full-text screening (Q1–3): Two persons separately - Data extraction (Q1–3): Standardized forms were developed and used; one person extracted, second person cross-checked the extraction - Critical appraisal (Q2): ROBIS [3] and single items of AMSTAR [4,5]; one person assessed risk of bias, second person cross-checked | - Recruitment: Project managers (contacted via e-mail and phone), managers forwarded information to teachers and students, interested persons were contacted by the study team via e-mail and phone - Questionnaire for project managers assessing characteristics of the project (e.g., learning goals, curricular and didactic characteristics) with single-/multiple-choice and open-ended questions - Standardized interviews assessing subjective effectiveness and feasibility, contextual factors during the project | - Recruitment: Members of the expert advisory board (expert interview) and cooperation partners (focus groups) were contacted via e-mail and phone - Semi-structured interview guide for expert interviews (30 minutes) (e.g., understanding of IPC, experiences in IPE, favourable factors influencing IPE) - Semi-structured interview guide for focus groups (120 minutes) (e.g., understanding of IPC, current IPE practice, expectations for the curriculum) |
| **Data analysis** | - Narrative summary using SPSS Version 22 (IBM) and Microsoft Excel 2019 | - Descriptive analysis using SPSS Version 22 (IBM) based on questionnaire and interview data from Microsoft Access 2019 - Qualitative content analysis of free-text answers [6] | - Structured content analysis [7] using MAXQDA Version 22 (VERBI) |

**References:**

1. Lüth F, Püschel L, von Gahlen-Hoops W, Balzer K, Rahn A. Curricula for interprofessional competencies in nursing education: Rapid review protocol [Internet]. In: OSF. 2022. https://osf.io/rhbvx/overview. Accessed 21 May 2026.
2. Kirkpatrick J, Kirkpatrick WK. An Introduction to the New World Kirkpatrick® Model. 2015. https://www.kirkpatrickpartners.com/wp-content/uploads/2021/11/Introduction-to-The-New-World-Kirkpatrick%C2%AE-Model.pdf. Accessed 21 May 2026.
3. Whiting P, Savović J, Higgins JPT, Caldwell DM, Reeves BC, Shea B, et al. ROBIS: A new tool to assess risk of bias in systematic reviews was developed. J Clin Epidemiol. 2016;69:225–34. doi: 10.1016/j.jclinepi.2015.06.005.
4. Shea BJ, Grimshaw JM, Wells GA, Boers M, Andersson N, Hamel C, et al. Development of AMSTAR: A measurement tool to assess the methodological quality of systematic reviews. BMC Med Res Methodol. 2007;7:10. doi: 10.1186/1471-2288-7-10/TABLES/2.
5. Shea BJ, Reeves BC, Wells G, Thuku M, Hamel C, Moran J, et al. AMSTAR 2: A critical appraisal tool for systematic reviews that include randomised or non-randomised studies of healthcare interventions, or both. BMJ. 2017;358:4008. doi: 10.1136/BMJ.J4008.
6. Sandelowski M. Whatever happened to qualitative description? Res Nurs Health. 2000;23:334–40. doi: https://doi.org/10.1002/1098-240X(200008)23:4<334::AID-NUR9>3.0.CO;2-G.
7. Kuckartz U, Rädiker S. Qualitative Inhaltsanalyse: Methoden, Praxis, Computerunterstützung: Grundlagentexte Methoden [Qualitative Content Analysis: Methods, Practice, Computer Support: Basic Texts Methods] (5th ed.). Weinheim: Beltz Juventa; 2022.
