# Supplementary material for: A longitudinal core curriculum to strengthen interprofessional competencies in German undergraduate nursing education: a mixed methods study
Source: BMC Med Educ. 2026 Sep 3;26:1449. doi: 10.1186/s12909-026-10273-z (PMC13560283; doi:10.1186/s12909-026-10273-z)
Supplement: Supplementary file 3 [file 12909_2026_10273_MOESM3_ESM.docx]

Additional file 3: Summary of main results of the design phase

| **Empirical approach** | **Mixed methods review on core curricula and recommendations for IPE (Interprofessional education) [1]** |
| --- | --- |
| **Included references** | N=18:   - Guidelines (n=2) - Frameworks (n=6) - Recommendations (n=4) - Core curricula (n=6) |
| **Main results** | The key aspects that were recommended and/or implemented/evaluated in the references included are summarized below:   - Learning outcomes: Understanding roles, interprofessional communication, collaboration and values, - Contents: interprofessional principles, ethics, evidence-based practice, patient safety - Frequently addressed professions: Nursing together with human medicine, physiotherapy, occupational therapy, social work, pharmacy (partly not exactly reported), - Didactic design: several modules/several related courses, mostly with group work, group discussions, case work, - Learning venues: often university/school and practice. - Contextual factors in implementation and evaluation:   - outer setting (education system): legal and political framework, existing networks of organizations,   - inner setting (Educational institutions): Further training opportunities, evaluation of IPE activities, cooperation partners and networks between vocational and academic training and practical institutions, group sizes in teaching, leadership culture, involvement of managers, learners and teachers as well as other stakeholders in the implementation of IPE activities, involvement of change agents/central coordination offices in the institutions, infrastructure, synchronization of timetables between training and study programmes, financial resources,   - inidividual domains: (stakeholder of the facilities): attitudes and competencies of those involved |
| **Limitations** | - The references included had to include the nursing profession, which limited the number of references included, - The recommendations/curricula had to cover a duration of at least three semesters (longitudinal character). |
| **Main**  **Conclusions** | - Interprofessional competencies should be integrated longitudinally into framework/core curricula to promote competencies for interprofessional collaboration, - Establishing national and international networks to promote IPE is important for the development and implementation of IPE, - Diverse, interactive learning methods in small groups and larger lectures are central to skills development, - So far, few curricula and recommendations cover several years of training. Many curricula/recommendations cover a maximum of one year of training. |

| **Empirical approach** | **Overviews of reviews [1]** |
| --- | --- |
| **Included references** | N=16:   - Systematic reviews (n=12) - Scoping review (n=1) - Integrative review (n=2) - Realist review (n=1) |
| **Main results** | - Frequently addressed competencies include interprofessional collaboration and communication as well as the understanding of roles. - Learning activities with nursing profession often take place with learners of human medicine, pharmacy, physiotherapy or occupational therapy. - The learning opportunities usually address the task areas of direct care practice (e.g. assessment, diagnostics or therapy of various care situations) or overarching tasks (e.g. communication). - The third learning venue (e.g. simulation) and the learning methods of simulation and direct involvement of people in need for care and caregivers were examined particularly frequently. - The evaluation of the learning opportunities is primarily based on self-reported measurements (e.g. questionnaires) and shows results in favour of the IPE activity and indifferent results at Kirkpatrick level 2 (knowledge, attitudes, skills) to level 4 (patient-relevant outcomes, among others). |
| **Limitations** | - The information in the reviews usually shows a high degree of aggregation of available information. - Many studies (included in the reviews) could be extracted, but they contain little meaningful evidence: only few studies used (randomized) parallel group comparisons, few studies on Kirkpatrick levels 3 (including professional behavior in [simulated] practice) and 4 (including patient- and organization-relevant outcomes), few studies used objective outcome measurement. - The evaluation of the learning opportunities was rarely carried out using robust evaluation methods. - The overall reporting quality of the included reviews is low (especially learning content, points in time in the training, measurement points of the evaluation). |
| **Main Conclusions** | - Indications of preferred characteristics (e.g. professions involved, reasons for action), - More robust evaluations (e.g. OSCE examinations) are required. |

| **Empirical approach** | **Mixed methods review of projects in German-speaking countries [1]** |
| --- | --- |
| **Included references** | N=21 national projects:   - Germany (n=19) - Switzerland (n=2) |
| **Main results** | - Learning opportunities with learners of nursing often take place together with learners of human medicine. Some studies also involve physiotherapy, speech therapy and social work learners. - Frequently used learning formats/methods are: problem-oriented learning, simulations, case work, discussions, lectures. - Interprofessional learning opportunities are offered in clinical or acute medical settings. - Most of the projects developed learning opportunities specifically for IPE in practice (interprofessional training wards), some learning opportunities are integrated into existing modules/learning units (anatomy, ethics), - Most projects use a mixed-methods approach for teaching evaluation, predominantly by means of pre-post measurement (often questionnaires, focus groups). - The survey mainly asks learners about the following target variables: satisfaction and feasibility of the course, learning gains, change in attitude towards other professional groups. - Reported contextual factors:   - Facilitating factors: participation in a practice-oriented setting, feedback and opportunities for reflection for learners, commitment of participants and a positive learning atmosphere,   - barriers: spatial separation, different study and examination regulations, stereotypes and role models of planners, lack of time resources. |
| **Limitations** | - A small sample size in almost all projects makes it difficult to generalize the results. - Possible influence of different prior experiences and abilities of learners and teachers as well as the institutional context. |
| **Main Conclusions** | - The results predominantly confirm the feasibility of the respective learning opportunities and a high level of subjective satisfaction reported by the learners, - Barriers at university and political level make implementation more difficult. - Most projects report the need for objective process evaluation and greater quantification of learning outcomes, as well as examination of the impact on healthcare practice. |

| **Empirical approach** | **Qualitative Interviews** |
| --- | --- |
| **Main results** | - Interprofessional competencies are often understood to mean the understanding of roles and interprofessional communication. - There is a wide variety of interprofessional topics/causes of action, with mobilization, basic principles, interprofessionalism, dealing with errors and interprofessional communication frequently mentioned. - Simulation-based education, internships and case work are often considered suitable methods. - Classroom teaching is advocated, although online teaching is also seen as expedient. - In addition to human, financial and time resources, a high level of commitment from all parties involved is considered particularly important. |
| **Limitationen** | - In one of the focus group interviews, it was not possible to obtain the perspective of the supervisors. - No learners were interviewed in expert interviews, so their perspectives could only be gathered in hierarchically composed focus group interviews. |
| **Main Conclusions** | Overall, interprofessional teaching was seen as an important component in the training of healthcare professions, but framework conditions are a limiting factor. Cooperation between educational institutions represents a major challenge in this respect. |

| **Empirical approach** | **Standardized Interviews** |
| --- | --- |
| **Main results** | - Frequently addressed competencies include interprofessional values, coordination and shared decision-making as well as reflection. - Learning opportunities with learners of nursing often take place together with learners of human medicine, physiotherapy, occupational therapy and speech therapy, predominantly in all three years of training. - Reported causes of action/topics:   - Direct care in orthopaedics, neurology, oncology, gastroenterology,   - Comprehensive tasks: person-centered communication, structured handover, ward rounds, discharge management - Learning methods used: skills training, case work, exercises with simulated patients, problem-based learning, structured debates, etc. Discussion formats, role play, practical learning, feedback/reflection, supervision. - Social forms of learning opportunities: lectures, seminars, exercises, communication training, project work, work shadowing. - Relevant contextual factors in planning and implementation:   - Facilitating factors: support from the school/course management, an additional project coordination position, staff training (especially training courses),   - Barriers: size of the university, (heterogeneous) curricular structures of the individual degree programs, different locations, low staffing levels, - Quantitative research and mixed-methods approaches were often used evaluation methods, mostly comprising (pre-post measurement without comparison group). Other data collection methods were controlled studies with a parallel comparison group and pre-post measurement as well as post-measurement only. |
| **Limitations** | For some projects, it was no longer possible to contact (former) learners, so that only little data is available from the learner perspective. |
| **Main Conclusions** | - National projects to promote interprofessional collaboration often address a small number of different professions. - The results show a predominantly high level of subjectively reported satisfaction among learners, teachers and project managers. Despite the frequently reported high organizational effort of interprofessional teaching-learning activities, the majority of the teachers and project managers rated the feasibility of their project as good. - The impact of the projects on care structures is predominantly considered to be low. The reasons cited are the lack of integration of the projects into the learning location practice and rigid structures of the care system outside the projects. |

| **Empirical approach** | **Expert workshop** |
| --- | --- |
| **Main results** | The learning opportunities should address all three learning venues and various healthcare professions as well as the people and caregivers to be cared for. The key elements developed are presented below, differentiated according to year of training (1st/2nd/3rd year of training) and academic training/vocational nursing training:  **Vocational nursing education**  1. year:   - Learning outcomes: interprofessional basics, understanding of roles, interprofessional communication and cooperation, - Learning content: dealing with errors, ethics, - Learning methods: seminars, job shadowing, group puzzles, learning workshops, peer learning, mentoring.   2. year:   - Learning outcomes: interprofessional communication, shared decision-making, reflection, understanding of roles, interprofessional values and cooperation, - Learning content: ethics, dealing with errors, interprofessional ward rounds, handover, support for family caregivers, - Learning methods: case work, simulation, digital formats, role play, involvement of people requiring care, project work, simulated patients, observation.   3. year:   - Learning outcomes: shared decision-making, professionall language, cooperation, reflection, - Learning content: patient safety, ethics, digitalization, advanced nursing practice, evidence-based practice, ward rounds, advice for patients and caregivers, error culture, - Learning methods: case work, involvement of people in need of care, digital formats, peer counselling, problem-based learning, job shadowing.   **Academic nursing education**  1./2. year:   - Learning outcomes: understanding roles, interprofessional principles and communication, - Learning content: Evidence-based practice, discharge management, ethics, chronic diseases, - Learning methods: role plays, simulation, work shadowing, problem-based learning, ward rounds.   3. year:   - Learning outcomes: further development of the understanding of roles, interprofessional communication, - Learning content: shared decision-making and target agreement, discharge management, evidence-based practice, - Learning methods: case work, digital methods, involvement of people in need of care and caregivers. |
| **Limitations** | One group discussion had to be cancelled due to last-minute cancellations of participants (including illness). Therefore, the group for year 1 and 2 (academic nursing education) were merged. |
| **Main Conclusions** | - The complexity of the teaching/learning situations/cases should increase in the course of the training. - Person-centred care forms a framework across the three years of training and should be addressed across the board. - Theory-practice transfer is important in all years of training. |

**Reference**

1. Lüth F, Püschel L, Leimer M, von Gahlen-Hoops W, Balzer K, Rahm AC. Konzeption interprofessioneller Curricula in der Pflegeausbildung: Wissenschaftsbasierte Empfehlungen für die Entwicklung und Umsetzung. [Designing Interprofessional Curricula in Nursing Education: Evidence-Based Recommendations for Development and Implementation]. In: Brühe R, von Gahlen-Hoops W, editors. Handbuch Pflegedidaktik II [Handbook of Nursing Education II]. Stuttgart: utb GmbH; 2024. p. 188–221. https://www.utb.de/doi/abs/10.36198/9783838562407-188-221. Accessed 21 May 2026.
