# Supplementary material for: A longitudinal core curriculum to strengthen interprofessional competencies in German undergraduate nursing education: a mixed methods study
Source: BMC Med Educ. 2026 Sep 3;26:1449. doi: 10.1186/s12909-026-10273-z (PMC13560283; doi:10.1186/s12909-026-10273-z)
Supplement: Supplementary file 4 [file 12909_2026_10273_MOESM4_ESM.docx]

Additional file 4: Examples of learning unit and module

| **Learning Unit 6** | **Deal constructively with errors in the interprofessional team**  “Error culture, that is already to be introduced as a topic … to make clear …, without good interprofessional collaboration there is a higher probability of not dealing well with such situations” (E10, pos. 186–189). | | |
| --- | --- | --- | --- |
| **Curricular classification** | Second year of training | Location of the expert commission according to para. 53 PflBG (nursing profession act) | Curricular unit 06 |
| **Scope** | **Learning Venue**   - **Nursing School:** 12 hrs* (hours) - **Clinical Practice:** 4 hrs - **Third Learning Venue:** 8 hrs - **Self-Directed Learning (SDL):** 4 hrs - **Total Workload:** 28 hrs   *1 hrs. = 45 minutes | | |
| **Possible**  **professions** | - Nursing - Medicine - Other health professions | | |
| **Tasks** | - Patient safety - Positioning within the interprofessional team | | |
| **Intentions and relevance** | Errors are part of everyday practice, yet they are often negatively associated and perceived as a personal failing. In healthcare, errors can have a wide range of consequences, potentially compromising patient safety.  Therefore, a constructive approach to dealing with errors is essential – one that focuses on identifying the root causes and developing strategies for error prevention and reduction.  Most errors result from human factors. It is therefore critical for all healthcare professionals to be aware of the risks associated with human fallibility, to address errors without assigning personal blame, and to actively implement strategies aimed at reducing errors. | | |
| **Competencies** | **Learners ...**   - are aware of the impact of errors on a personal level and in the context of patient safety - recognize interprofessional collaboration as a key factor in improving patient safety - analyze the various areas of responsibility within the interprofessional team and the shared goal of patient safety - jointly analyze professional situations to identify potential sources of error - analyze interprofessional conflicts in the context of errors and their origins, and reflect on the differing perspectives of the professions involved - apply crisis resource management (CRM) principles within the interprofessional team - communicate effectively within the interprofessional team to prevent errors - reach joint decisions in relation to (potential) errors - engage in constructive discussions about error situations within the interprofessional team and derive consequences for future collaboration - collaboratively develop preventive measures to reduce errors - deepen their awareness of differences in professional terminology | | |
| **Content** | - Error theories (e.g., Reason) - Sources of error - Importance of patient safety and consequences of errors - Personal impact of errors - Second victim phenomenon - Principles of crisis resource management (CRM) - Typical topics in the context of errors (e.g., medication safety) | | |
| **Learning methods**  **(recommendations)** | **Nursing School:**  Part 1 (self-directed learning, SDL):   - Basic principles of communication (e.g., Schulz von Thun, Riemann-Thomann model)   Part 2:   - Short presentation on error theory/theories, patient safety and human factors; with optional reference to error reporting systems (e.g., Critical incident Reporting System) - Anonymous survey (e.g., via Mentimeter or similar) on personal experiences with making mistakes - Media – Film: Just a Routine Operation (optional) - Think-Pair-Share:   - Individual reflection on an error (not necessarily professional); focus areas: emotional response, personal handling of the error, possible reactions from other people/professions, personal consequences   - Exchange reflections with a partner (ideally interprofessional)   - Interprofessional small group: brief presentation of error situations, joint analysis, and development of preventive strategies for error reduction   - Presentation of results using posters - Gallery walk to present group results (posters)   Part 3 (after completing the observation task at the clinical practice):   - Reflect on the results of the observation task (1 or 2) - Develop preventive measures for identified sources of error (e.g., Lotus Blossom Technique, Disney Method) - Discuss error situations (exercise, optionally as SBL)   **Clinical Practice:**   - Learning task 1: Observation – identify sources of error in professional practice - Learning task 2: Observation – describe error situations (how did the individuals involved respond?)   **Third Learning Venue:**  Simulation on medication safety | | |
| **Suggested case scenarios** | Cases provided by learners and/or cases from observation tasks,  simulation-based scenario | | |
| **Methods for documenting and reflecting on results** | - Poster presentation with gallery walk (results from Think-Pair-Share activity) - Formative assessment of simulation | | |
| **References** | Borgwart, J., Kolpatzik, K. (Eds.) (2010). Aus Fehlern lernen – Fehlermanagement in Gesundheitsberufen. [Learning from mistakes – error management in healthcare professions]. Berlin, Heidelberg: Springer.  Pfeilschifter, W., Rall, M. (2022). Optimierung der Teamarbeit auf der Stroke Unit durch Crew Resource Management und Simulations-Teamtraining zur Verbesserung der Patientensicherheit und Mitarbeiterzufriedenheit. [Optimisation of teamwork in the stroke unit through crew resource management and simulation team training to improve patient safety and employee satisfaction].In: Cassier-Woidasky, A.K., Pfeilschifter, W., Glahn, J. (Eds.). Pflege von Patienten mit Schlaganfall. [Caring for stroke patients]. 3rd ed. Kohlhammer.  Rall, M. (2022). Sicherheit trotz Fehlern: Crew Resource Management (CRM) für Medizinische Einsatzteams (MET). [Safety despite errors: Crew resource management (CRM) for medical emergency teams (MET)]. In: Koch, T., Heller, A. R., Schewe, J.C. (Eds.). Medizinische Einsatzteams. [Medical response teams]. Berlin, Heidelberg: Springer. 75–86.  Reason J. (2000). [Human error: models and management](http://www.ncbi.nlm.nih.gov/pubmed/10720363). *British medical journal* (18) 3:768–770. https://doi/ [10.1136/bmj.320.7237.768](https://doi.org/10.1136/bmj.320.7237.768).  St. Pierre, M., Hofinger, G. (2020). Human Factors und Patientensicherheit in der Akutmedizin. [Human factors and patient safety in acute medicine]. 4th ed. Berlin: Springer. https://doi.org/10.1007/978-3-662-60485-4  Thomas, V. (2021). Fehlermeldeverhalten in der Pflege. [Error reporting behaviour in nursing care]. Springer. https://doi.org/10.1007/978-3-658-33675-2. | | |
| **Contextual factors and implementation tips** | - Qualifications of instructors: Bachelor's or master's degree in nursing education or equivalent qualification - Mandatory cooperation between various training institutions and universities - Theoretical content may be delivered partially via virtual formats - Simulation: Minimum of two instructors, at least one with a master's degree in nursing education (technician may be involved if needed) - Clinical facilities may be used as a simulation setting - Instructors have completed training in simulation-based teaching - Small group composition: Consider varying levels of professional experience and prior knowledge | | |

| **Module title** | **We as an interprofessional team …**  “understand what our own role is … it is not always just about oneself, but also about one’s position within the team” (E7, 411–418). | |
| --- | --- | --- |
| **Curricular classification** | First year of training | |
| **Scope** | **Seminar: 1 semester hours**  **Exercise: 1 semester hours**  **Practical training: 0.5 semester hours** | **Workload:**  30 hrs* in-person study  40 hrs self-directed learning  20 hrs practical training  *1 hrs. = 45 minutes |
|  | Credits: 3 credit points | |
| **Possible**  **professions** | - Nursing - Medicine (first/second year) - Physiotherapy (first year) - Optional: speech therapy, occupational therapy | |
| **Tasks** | - Mobility enhancement - Roles and responsibilities within the interprofessional team | |
| **Intentions and relevance** | During their first orientation internship with a practical training partner (in acute inpatient, outpatient, or long-term care settings), nursing students encounter a range of health professions involved in the care of person in need of care; optimal care through interprofessional collaboration is a crucial but not self-evident goal.  Additionally, students are introduced early on to hands-on, body-related activities.  The focus of this module is on developing an understanding of the individual’s professional role as well as the roles and responsibilities of other health professions. Students are encouraged to view their future profession within the context of interprofessional collaboration. They should also recognize that interprofessional teams share common goals, knowledge bases, and principles of evidence-based practice.  The promotion of mobility is a common content for observation, shared experiences and reflection on professional knowledge, tasks, and activities. | |
| **Competencies** | **Students ...**   - **explore and compare the roles and responsibilities of their own profession with those of other academic and non-academic health professions involved in the care of individuals with varying care needs, with a particular focus on promoting mobility.** - identify and describe similarities and differences in the roles, responsibilities, and relevant bodies of knowledge of the participating professions - engage in dialogue with other health professions to identify common stereotypes and biases associated with the participating professions, and research strategies to counteract them - communicate their own role and areas of responsibility clearly and in an understandable manner when interacting with person in need of care, families, and other health professionals - are sensitized to the cross-professional relevance of evidence-based practice principles, for example, in the context of enhancing mobility - respectfully acknowledge the values, rights, cultures, responsibilities, and professional expertise of other professions, especially medicine and physiotherapy - recognize, reflect on, and respect the boundaries of their own area of responsibility in relation to those of other professions - understand the importance of interprofessional collaboration in the care of individuals with nursing needs and analyze the principles of teamwork | |
| **Content** | - Interprofessional basics: Definition of Interprofessional education (IPE) and IPP (Interprofessional practice) (domains of interprofessional collaboration), areas of responsibility of the various professions - Basics for promotion of mobility: Knowledge of the anatomy and physiology of the musculoskeletal system, the significance of mobilityt for health, quality of life, and participation, the relationship between mobility and care dependency, assessment of mobility, strategies and techniques to support movement in individuals with limited mobility, including evidence-based recommendations and decision-making tools | |
| **Learning methods**  **(recommendations)** | **University:**   - Interprofessional basics: Perspective change, interprofessional escape game (card-based activity) (2 TU (teaching unit, 1 TU=45 minutes)) - Seminar: Fundamentals of interprofessional collaboration (5 TU) - Seminar: In-depth exploration of roles, responsibilities, similarities, and differences within the interprofessional team (3–5 TU) - Seminar: Joint problem-solving of potential challenges in mobility and mobility enhancement from an interprofessional perspective (3–4 TU)   **Clinical practice:**  Internship: Interprofessional shadowing (with a healthcare professional from another health discipline, minimum three years of practical experience) (8 TU)  **Third learning venue:**  Exercise: Case-based planning, implementation, evaluation, and reflection of interprofessionally coordinated nursing and treatment processes (with flexible focus on assessment and interventions to enhance mobility) on mobility enhancement (preferably as a block course)   - Introduction (1 TU, all participants) - Case analysis and assessment (with simulated persons) (3 TU, in small groups) - Planning and implementation, including use of practical skills (4 TU, with simulated patients) - Evaluation and reflection (2 TU) - Presentation and discussion of results (4 TU, all participants) | |
| **Required media/materials** | - **Handouts for seminar sessions:** Interprofessional evidence-based practice, IPE and IPP, mobility and promotion of mobility - **Case-based planning guide for skills training** - **Practice assignment for reflecting on existing structures of interprofessional collaboration** - **Shadowing report** | |
| **Suggested case scenarios** | **Case 1:**   - Setting: Acute care hospital, neurology ward - person in need of care: Mr./Ms. Meisen, height: 1.80 m, weight: 75 kg - Medical condition: Acute ischemic stroke of the A. cerebri media with left-sided hemiparesis - Intervention: Mobilization from bed - Profession-specific focus areas (discussed interprofessionally):   - Assessment tools (e.g., motor function, self-care abilities)   - Diagnoses/diagnostic criteria (e.g., related to mobility)   - Treatment/care goals (based on previous diagnoses)   - Appropriate interventions (e.g., mobility-enhancing activities, assistive device counseling, support for mobility and other self-care needs)   - Evaluation criteria (based on defined care goals, e.g., safe transfer from bed to chair and vice versa)   **Case 2:**   - Setting: Long-term care facility - person in need of care: Ms./Mr. Waldner, no close relatives except for one cousin (living 500 km away) - Medical condition: Born with cerebral palsy; limited mobility (uses forearm crutches, occasionally requires prompts for mobilization from bed) - Interventions: Mobilization from bed and use of forearm crutches - Profession-specific focus areas:   - Assessment tools (e.g., motor function, self-care abilities)   - Diagnoses/diagnostic criteria (e.g., related to mobility and swallowing disorders)   - Treatment/care goals (based on previous diagnoses)   - Appropriate interventions (e.g., mobility-enhancing activities, assistive device counseling, support for mobility, and other self-care needs)   - Evaluation criteria (based on defined care goals, e.g., safe transfer from bed to chair and vice versa) | |
| **Methods for documenting and reflecting on results** | **Possible formative assessments (“Assessment for Learning”):**   - Twenty-minute interview with a professional following the shadowing experience - Interprofessional one-minute paper after each teaching session - Collaborative document on the added value of interprofessionalism (for the learner, for the learner’s own discipline, for science, for society, or for the team), updated within the interprofessional group after each seminar block - Interprofessional learning journal   **Possible summative assessment (“Assessment of Learning”):**   - Joint case documentation during skills training | |
| **References** | Barr, H., Gray, R., Helme, M., Low, H., & Reeves, S. (2016). Interprofessional Education Guidelines 2016. Available from: https://www.caipe.org/resources/publications/caipe-publications/barr-h-gray-r-helme-m-low-h-reeves-s-2016-interprofessional-education-guidelines  Behrens, J., & Langer, G. (2022). Evidence-Based Nursing and Caring. Bern: Hogrefe.  Dow, A.W., DiazGranados, D., & Baernholdt, M. (2021). Interprofessional Practice and Education. In: Baernholdt, M. & Boyle, D.K. (Eds.). Nurses Contributions to Quality Health Outcomes. Cham: Springer Nature Switzerland AG.  Mangold, S. (2013). Evidenzbasiertes Arbeiten in der Physio- und Ergotherapie: Reflektiert - systematisch - wissenschaftlich fundiert. [Evidence-based work in physiotherapy and occupational therapy: Reflected – systematic – scientifically sound]. Springer.  Meier, O., Kleinberger, U. & Gerber-Grote, A. (2022). Interprofessionalität – ein gesundheitswissenschaftlicher Begriff aus dem Blickwinkel der Linguistik. [Interprofessionalism – a health science term from the perspective of linguistics] *International Journal of Health Professions*, 9(1), 139–150.  Pundt, J., & Kälble, K. (Eds.) (2015). Gesundheitsberufe und gesundheitsberufliche Bildungskonzepte. [Health professions and health profession education concepts]. Bremen: APOLLON University Press.  Schlesselmann, E. (Eds.) (2019). Bewegung und Mobilitätsförderung. [Movement and mobility promotion]. (1st ed.). Bern: Hogrefe.  World Health Organization (WHO) (2010). Framework for Action on Interprofessional Education & Collaborative Practice. Geneva. http://whqlibdoc.who.int/hq/2010/WHO_HRH_HPN_10.3_eng.pdf | |
| **Contextual factors and implementation tips** | Prerequisites:  Basic knowledge of care processes and the responsibilities of the respective health professions  Collaborations:   - Practice partners for shadowing placements/practical training - Degree programs in the health professions   Qualifications of instructors and student tutors:  In accordance with the minimum qualification requirements as stipulated by higher education regulations | |
